# Pre-Treatment Hemoglobin Concentration and Absolute Monocyte Count as Independent Prognostic Factors for Survival in Localized or Locally Advanced Prostate Cancer Patients Undergoing Radiotherapy

**DOI:** 10.3390/biomedicines10102514

**Published:** 2022-10-08

**Authors:** Łukasz Magrowski, Oliwia Masri, Jakub Ciepał, Gabriela Depowska, Zuzanna Nowicka, Rafał Stando, Krystyna Chimiak, Gabriela Bylica, Barbara Czapla, Małgorzata Masri, Franciszek Cichur, Iwona Jabłońska, Marta Gmerek, Piotr Wojcieszek, Tomasz Krzysztofiak, Jacek Sadowski, Rafał Suwiński, Paweł Rajwa, Matthias Moll, Gregor Goldner, Wojciech Majewski, Marcin Miszczyk

**Affiliations:** 1 IIIrd Department of Radiotherapy and Chemotherapy, Maria Sklodowska-Curie National Research Institute of Oncology, 44-102 Gliwice, Poland; 2 Department of Biostatistics and Translational Medicine, Medical University of Lodz, 90-419 Lodz, Poland; 3 Department of Radiotherapy, Holy Cross Cancer Center, 25-734 Kielce, Poland; 4 Brachytherapy Department, Maria Sklodowska-Curie National Research Institute of Oncology Gliwice Branch, 44-102 Gliwice, Poland; 5 II Clinic of Radiotherapy and Chemotherapy, Maria Sklodowska-Curie National Research Institute of Oncology, Gliwice Branch, 44-102 Gliwice, Poland; 6 Department of Urology, Comprehensive Cancer Center, Vienna General Hospital, Medical University of Vienna, Währinger Gürtel 18-20, 1090 Vienna, Austria; 7 Department of Urology, Medical University of Silesia, 3-go Maja 13-15, 41-800 Zabrze, Poland; 8 Department of Radiation Oncology, Comprehensive Cancer Center, Medical University of Vienna, Währinger Gürtel 18-20, 1090 Vienna, Austria; 9 Radiotherapy Department, Maria Skłodowska-Curie National Research Institute of Oncology, Wybrzeże Armii Krajowej 15, 44-102 Gliwice, Poland

**Keywords:** hemoglobin, monocytes, overall survival, prostate cancer, radiotherapy

## Abstract

The prognostic value of inflammatory indices, such as the absolute monocyte count (AMC), has been a subject of interest in recent prostate cancer (PCa) studies, while hemoglobin concentration (HGB) has been recognized as a survival factor in castration-resistant metastatic prostate cancer, but its value remains unclear in localized diseases. The aim of this study was to test the prognostic value of these two simple and inexpensive biomarkers for survival and was based on a cohort of 1016 patients treated with primary radiotherapy and androgen deprivation therapy for localized or locally advanced intermediate- or high-risk PCa. Complete survival data were available for all cases and were based on the National Cancer Registry, with a median observation time of 120 months (Interquartile Range (IQR) 80.9–144.7). Missing blood test data were supplemented using the Nearest Neighbor Imputation, and the Cox Proportional Hazards Regression model was used for analysis. The median age was 68.8 years (IQR 63.3–73.5). The five-year overall survival was 82.8%, and 508 patients were alive at the time of analysis. The median time between blood tests and the first day of radiotherapy was 6 days (IQR 0–19). HGB (*p* = 0.009) and AMC (*p* = 0.003) were independent prognostic factors for survival, along with age, Gleason Grade Group, clinical T stage and maximum prostate-specific antigen concentration. This study demonstrates that HGB and AMC can be useful biomarkers for overall survival in patients treated with radiotherapy for localized intermediate- or high-risk PCa.

## 1. Introduction

There are a range of therapeutic options available for the treatment of localized prostate cancer (PCa). However, it has been shown that it is unlikely that patients will experience a survival benefit from upfront interventional treatment for localized prostate cancer within 10 years of diagnosis [1], and, in general, asymptomatic patients with a life expectancy below five years are discouraged from seeking interventional treatment methods [2]. Therefore, an adequate estimation of a patient’s expected survival, based on the WHO’s Life Tables [3] or the Memorial Sloan Kettering Male Life Expectancy Tool [4], for example, is crucial for an individualized and patient-tailored approach when choosing the appropriate treatment strategy. The Charlson Comorbidity Index or its ‘Prostate Cancer Specific’ modification are the most widely used tools to stratify mortality risk in PCa patients [5,6,7]. In situations where the expected survival is unclear, other prognostic factors are highly recommended.

Hemoglobin concentration (HGB) is a routinely measured blood parameter, and its prognostic value is well-recognized across various malignancies [8,9]. The prognostic value of HGB in metastatic prostate cancer has also been documented [10,11,12]. However, only few studies thus far have evaluated the ability of HGB to predict outcomes in localized PCa [13,14].Considering its value in predicting both disease-specific mortality [10] and all-cause mortality [15,16], HGB promises to be a valuable tool to predict the survival of PCa patients.

Tumor-associated macrophages (TAM) are differentiated circulating monocytes in the tumor site and have been reported to promote tumor genesis and progression [17]. TAM, which correlates with absolute monocyte count (AMC) [16], has proven to be a prognostic factor for overall survival in PCa patients [15]. Therefore, routinely measured AMC may prove to be a useful prognostic factor for survival in PCa patients, as suggested in recent publications [18,19].

This study aims to analyze the value of HGB and AMC as independent prognostic factors for overall survival (OS) and freedom from distant metastases (FFDM) in patients treated with radiotherapy for localized and locally advanced intermediate- or high-risk PCa.

## 2. Materials and Methods

### 2.1. Patients

**Figure 1:**
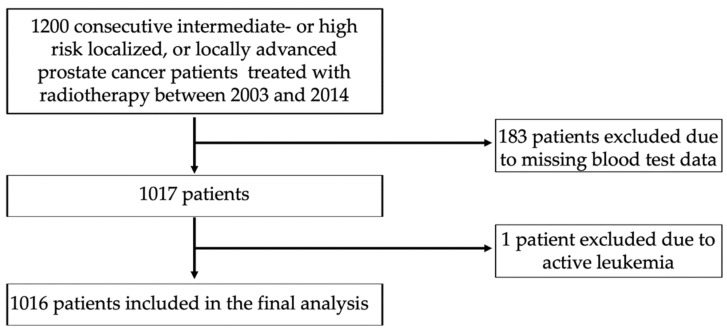
Flow chart of the study population.

From February 2003 to November 2014, 1200 consecutive patients underwent radical radiotherapy at a single tertiary center for histologically proven localized or locally advanced (T_1c_-T_4_, N_0_/N_1_, M_0_) intermediate- or high-risk prostate cancer. A total of 184 cases were excluded due to a lack of pre-irradiation blood tests (*n* = 183) or a co-existing leukemia (*n* = 1), and 1016 patients were included in the final analysis (Figure 1). Tumor staging was assessed retrospectively according to the 2017 Union for International Cancer Control’s 8th edition classification [20] based on the available results from digital rectal examinations; transrectal ultrasonography; bone scintigraphy; computed tomography (CT) or magnetic resonance imaging (MRI) of the pelvis and abdomen; chest radiography; and, in some cases, 18F-fluorocholine-PET, which was later superseded by PET-PSMA. All the tumors were confirmed histopathologically based on material obtained from a fine-needle biopsy or from a transurethral resection of the prostate. The International Society of Urological Pathology (ISUP) Gleason Grading Group [21] was assessed retrospectively based on the available Gleason score data. Blood parameters were collected from tests performed no later than two days after the start of the external beam radiotherapy (EBRT) or the first fraction of the brachytherapy boost (BT-boost), whichever occurred earlier. This study was approved by the bioethics committee of Maria Sklodowska-Curie National Research Institute of Oncology, Gliwice, Poland (approval no. KB/430-82/21), and patient agreement was waived due to the retrospective nature of this analysis.

### 2.2. Follow-up

Clinical follow-up data were collected retrospectively based on the patient’s medical records for FFDM and the Polish National Cancer Registry data for OS. The follow-up duration was calculated from the first day of radiotherapy. Follow-up visits were scheduled every 3 months in the first 1–2 years, every 6 months until 5 years after treatment and annually thereafter. In the event of a rising PSA level and a reasonable presumption of distant metastases, medical imaging was performed, including methods such as bone scintigraphy; 18F-fluorocholine or PSMA-PET; MRI and CT-scan.

### 2.3. Statistical Analysis

The primary endpoint was OS. The secondary endpoint was FFDM. Both endpoints were calculated from the first day of radiotherapy to the day of death or last known time point when the patient was alive for OS and the occurrence of distant metastases or last follow-up visit for FFDM. Nearest Neighbor Imputation was used to impute missing laboratory test values with K = 3 and was based on the values of available parameters. A summary of all the parameters before and after imputation is presented in Supplementary Table S1. Continuous variables were described using medians with interquartile ranges (IQR) due to the non-normality of the distribution and were verified using the Shapiro–Wilk test. Differences between the groups were assessed using the Mann–Whitney U test or Kruskal–Wallis test, depending on the number of groups, and associations between continuous variables were tested using the Spearman rank correlation test. The Cox Proportional Hazards model was used for survival analysis, and hazard ratios (HRs) and 95% confidence intervals (95% CIs) were reported. Laboratory parameters were selected for inclusion in the multivariate analysis based on their significance in univariate analysis, co-linearity and known prognostic value for prostate cancer. The Akaike Information Criterion (AIC) was used to evaluate the models. *p*-values lower than 0.05 were considered statistically significant, and all the tests were two-sided. All calculations were performed using Statistica 13.3 software by StatSoft (TIBCO Software, Palo Alto, CA, USA) [22].

## 3. Results

### 3.1. Treatment and Patient Outcomes

Patients were treated with EBRT or EBRT combined with a high-dose-rate BT-boost in 192 (18.9%) cases. There were 14 patients with metastasis in a single regional lymph node (N_1_). These patients were given a boost with irradiation. Pelvic lymph node irradiation was performed in 76% (*n* = 772) of patients, up to a total dose of 44–50 Gy in 2 Gy fraction doses. Detailed data on irradiation doses are described in Supplementary Table S2. The majority of patients (*n* = 953) received neoadjuvant ADT (Neo-ADT), in most cases based on the gonadotropin-releasing hormone agonist (GnRH) combined with a nonsteroidal anti-androgen drug (NSAA) (85%, *n* = 810). The GnRH agonist was used as a monotherapy in 116 cases (12.2%), and NSAA was used in 31 cases (3.3%). The median duration of Neo-ADT was 4.6 months (IQR 3.2–7), and the median total duration of ADT was 28.6 months (IQR 14.9–41.9). The median time from the blood tests to the first day of EBRT or BT-boost was 6 days (IQR 0–19). Detailed patient and treatment characteristics are described in Table 1.

**Figure 2:**
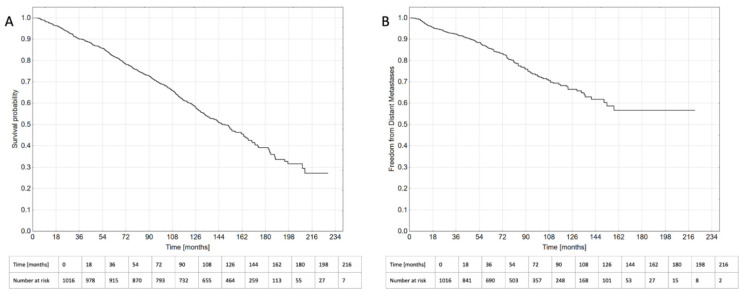
Overall survival (**A**) and freedom from distant metastases (**B**) in patients treated with radiotherapy for localized or locally advanced prostate cancer.

Median follow-up was 120 months (IQR 80.9–144.7) for OS and 57.4 months (IQR 30.3–97.4) for FFDM. Five-year overall survival was 82.8%, and 508 (50%) patients were alive at the date of analysis (Figure 2A). Distant metastases occurred in 177 (17.4%) cases (Figure 2B). The main metastatic sites were bones (*n* = 96) and lymph nodes (*n* = 40) or both (*n* = 23). The metastatic spread was diagnosed in the majority of cases with bone scintigraphy (*n* = 58), 18F-fluorocholine-PET (*n* = 53), CT-scan (*n* = 33), PSMA-PET (*n* = 12) or MRI (*n* = 11). A second malignancy was diagnosed during follow-up in 81 patients, including 28 cases of colon cancer, 13 cases of lung cancer, and 9 cases of non-melanoma skin cancer.

### 3.2. Predicting Overall Survival Based on Clinical Variables and Blood parameters

In the univariate analysis (UVA) age, the ISUP Grade Group, clinical T stage, Eastern Cooperative Oncology Group Performance Status (ECOG), RT modality and maximum PSA concentration (mPSA) were significant for OS, along with HGB, NLR, WBC, NEUT, AMC, EO, RBC, HCT and RDW (Table 2). Several moderate to strong correlations were observed between blood parameters (Supplementary Table S3), and based on the significance in UVA, known clinical relevance and collinearity with other predictors of HGB and AMC were included in the multivariate analysis (MVA) model for survival prediction.

HGB (*p* = 0.009), AMC (*p* = 0.003), age (*p* < 0.001), clinical T stage, ISUP Grade Group and mPSA (*p* = 0.021) remained significant predictors for OS in MVA (Table 2). Adding HGB and AMC to the model reduced the AIC to 6102.68 as compared to 6111.54 for the model with clinical prognostic factors alone. 

### 3.3. Predicting Freedom from Distant Metastases Based on Clinical Factors and Blood Parameters

In UVA, the ISUP Grade Group, clinical T stage, ECOG, RT modality, mPSA HGB and AMC were significant prognostic factors for FFDM (Table 3). Despite the lack of significance in UVA, age was included for the MVA because of its known prognostic value. In MVA, only the ISUP grade group, clinical T stage and RT modality remained significant (Table 3).

### 3.4. Hemoglobin and Monocyte Association with Prognostic Factors

An exploratory analysis was conducted to investigate the association of HGB and AMC with relevant clinical factors. HGB was not associated with ECOG (*p* = 0.592), RT modality (*p* = 0.982), ISUP Grade Group (*p* = 0.576) or clinical T stage (*p* = 0.075); however, it was correlated weakly with patient age (*p* < 0.001, *R* = −0.164), duration of Neo-ADT (*p* < 0.001, *R* = −0.144) and mPSA (*p* = 0.036; *R* = −0.067).

AMC was not correlated with patient age (*p *= 0.152, *R* = 0.045), mPSA (*p* = 0.135, *R* = 0.047) or duration of Neo-ADT (*p* = 0.952, *R* = 0.002) and was not associated with clinical T stage (*p* = 0.071). There was a positive association between AMC and BT-boost for RT modality (*p* < 0.001), an ECOG score of 1 or 2 (*p* = 0.035) and ISUP Grade Group (*p* = 0.004).

## 4. Discussion

Unnecessary treatment of PCa patients whose life expectancy is insufficient for the treatment to have a noticeable impact on their survival can be associated with the risk of side effects and can significantly reduce the patient’s quality of life [23,24]. A personalized approach to the treatment of each patient requires a proper estimation of their life expectancy, especially if aggressive treatment is planned. This publication has shown that HGB and AMC may provide additional information about the expected survival of patients with PCa in daily practice. Most importantly, this study showed that HGB and AMC contributes independent prognostic information for OS.

### 4.1. Hemoglobin Concentration

The literature data relating to the prognostic value of HGB in localized or locally advanced PCa are very limited and have shown mixed results. D’Amico et al. [25] found that ADT-related decline in pre-treatment HGB resulted in an increased risk of biochemical failure, but OS was not assessed as an endpoint. Parker et al. failed to reproduce these results [14]. Pai et al. analyzed the relationship between the pre-treatment HGB level and survival in PCa patients undergoing EBRT but were unable to find a significant association with OS or biochemical control [13]. These studies focused on ADT-induced anemia, which translated into reduced tumor oxygenation, which was expected to result in worse local control and an increased risk of biochemical failure. However, ADT can lead to improvements in tumor vascularization, which could improve oxygenation [26]. In our study, 93.8% of patients received Neo-ADT and its duration had a weak correlation with HGB (*R* = −0.144). Therefore, its potential impact on the results seems to be limited.

The phenomenon of pretreatment HGB association with PCa patient survival could be explained by several different hypotheses, including the above-mentioned ADT-induced anemia. It is highly probable that a patient’s general condition is reflected, in part, by the HGB level because many diseases are known to influence either the total amount of HGB or the ability of molecules to bind oxygen at the same partial pressure of oxygen [27]. Unfortunately, in this study, it was impossible to collect reliable data on the patients’ general condition and comorbidities, which would be sufficient to reach a conclusion in the analysis. It is also possible that HGB, through its association with patients’ general condition, or with the primary tumor itself, reflects the body’s subclinical ability to eliminate metastatic cells [28]. Since the proportion of patients with known distant metastases in our study population accounted for 26.4% of total deaths, the association of HGB with the occurrence of distant metastases could partially explain its relationship with OS. However, HGB was a significant parameter in UVA for FFDM (HR 0.852; 95% CI 0.745–0.9744; *p* = 0.0192), but it was not significantly associated with FFDM after controlling for clinical variables. Taking this analysis into account, the most plausible hypothesis is likely to be the product of both HGB association with patients’ general condition and PCa severity.

### 4.2. Absolute Monocyte Count

Hayashi et al. [18] found that AMC can predict adverse pathological features and the risk of postoperative biochemical failure. The authors reported a significant correlation between AMC and TAM in tumor sites. AMC has been shown to be a prognostic factor for both cancer-specific survival and OS in a large retrospective analysis by Wang et al. [19]. In our study, TAM presence in the tumor site was not analyzed; however, AMC was found to be higher in patients with a higher ISUP Grading Group (*p *= 0.004), as previously reported by Hayashi et al. [29]. Some authors have suggested that a predictive value of AMC is related to TAM [30,31], which was reported to be associated with progression in various malignancies [17,29]. In this study, AMC was associated with FFDM in UVA, but not in the MVA. This was likely due to the inclusion of ISUP Grade Groups. However, AMC remained an independent prognostic factor for patient survival. This suggests that AMC association with survival is more complex than just its correlation with tumor pathological adverse features. Further studies are highly warranted.

### 4.3. Limitations

The main limitations of this study are its retrospective character, missing comorbidity and smoking data. This study does not include low-risk patients, for whom survival length estimation during treatment strategy planning could be especially important. The Neo-ADT used for the majority of patients could be a confounding factor in the interpretation of pre-treatment blood tests, and pre-ADT data could prove to be more useful in this scenario. While all the patients were treated with EBRT, the wide range of RT modalities and fractionation schemes could have influenced the results.

## 5. Conclusions

Hemoglobin concentration and absolute monocyte count are simple and inexpensive biomarkers that are associated with survival in patients treated for intermediate- or high-risk localized or locally advanced prostate cancer and can improve patient-tailored treatment decision making.

## Figures and Tables

**Table 1:** Baseline patient characteristics.

Parameter	Study Group *N =* 1016
Age (median) [years]	68.8 (IQR 63.2–73.5)
ECOG	
0	79.3%
1	20.5%
2	0.2%
NCCN Risk Group	
Favorable intermediate	6.3%
Unfavorable intermediate	23.9%
High	45.7%
Very high	24.1%
ISUP Grade Group	
1	38.8%
2	29.5%
3	12.5%
4	8.9%
5	8.2%
Missing data	2.2%
Clinical T stage	
T1c	35.8%
T2a	11.7%
T2b	18.7%
T2c	17.2%
T3a	9.8%
T3b	5.5%
T4	1.2%
Pre-radiation PSA (median) [ng/mL]	0.6 (IQR 0.11–3.42)
PSA density (median) [ng/mL^2^]	0.64 (IQR 0.33–1.14)
mPSA (median) [ng/mL]	24.39 (IQR 13.28–41.99)
mPSA	
<10 ng/mL	16%
≥10 ng/mL, <20 ng/mL	21.7%
≥20 ng/mL	61.1%
Missing data	1.2%
TURP	5.8%
Neo-ADT	93.8%
Duration of Neo-ADT (median) [months]	4.6 (IQR 3.2–7)
Adjuvant ADT	86.8%
Total duration of ADT (median) [months]	28.6 (IQR 14.9–41.9)
Radiation modality	
EBRT	81.1%
EBRT + single BT-boost	12.3%
EBRT + double BT-boost	6.6%
Lymph node irradiation	76%
NLR (median)	1.92 (IQR 1.42–2.62)
PLR (median)	114.8 (IQR 90.1–145)
LMR (median)	3.32 (IQR 2.57–4.28)
WBC (median) [10^3^/μL]	6.43 (IQR 5.3–7.7)
LYMPH (median) [10^3^/μL]	1.86 IQR 1.5–2.35)
NEUT (median) [10^3^/μL]	3.61 (IQR 2.87–4.56)
AMC (median) [10^3^/μL]	0.56 IQR 0.45–0.71)
EO (median) [10^3^/μL]	0.15 (IQR 0.09–0.22)
BASO (median) [10^3^/μL]	0.03 (IQR 0.02–0.04)
RBC (median) [10^6^/μL]	4.48 (IQR 4.2–4.77)
HGB (median) [g/dL]	13.8 (IQR 13–14.6)
HCT (median)	40.6% (IQR 38.7–42.9)
RDW (median)	13.4% (IQR 12.8–14)
PLT (median) [10^3^/μL]	211 (IQR 179–249.5)
PDW (median) [fL]	12.3 (IQR 11.2–13.6)

NCCN—National Comprehensive Cancer Network, ISUP—International Society of Urological Pathology, PSA—prostate-specific antigen, mPSA—maximum PSA concentration, TURP—transurethral resection of the prostate, ADT—androgen deprivation therapy, EBRT—external beam radiation therapy, BT-boost—brachytherapy boost, RT—radiotherapy, NLR—neutrophil-to-lymphocyte ratio, PLR—platelet-to-lymphocyte ratio, LMR—lymphocyte-to-monocyte ratio, WBC—absolute white blood cell count, LYMPH—absolute lymphocyte count, NEUT—absolute neutrophile count, AMC—absolute monocyte count, EO—absolute eosinophile count, BASO—absolute basophile count, RBC—absolute red blood cell count, HGB—hemoglobin concentration, HCT—hematocrit, RDW—red blood cell distribution width, PLT—absolute platelet count, and PDW—platelet distribution width.

**Table 2:** Cox Proportional Hazards Regression Analysis for overall survival in patients treated with radiation therapy for localized or locally advanced prostate cancer.

Variable	Univariate Analysis		Multivariate Analysis	
	Hazard Ratio (95% CI)	*p*-Value	Hazard Ratio (95% CI)	*p*-Value
HGB [g/dL]	0.853 (0.789–0.922)	**<0.001**	0.899 (0.83–0.975)	**0.009**
AMC [10^3^/μL]	2.216 (1.497–3.282)	**<0.001**	1.918 (1.243–2.959)	**0.003**
Age [years]	1.067 (1.052–1.082)	**<0.001**	1.065 (1.05–1.081)	**<0.001**
ISUP Grade Group				
2 vs. 1	1.296 (1.042–1.611)	**0.019**	1.215 (0.970–1.523)	0.089
3 vs. 1	1.287 (0.963–1.72)	0.088	1.140 (0.846–1.538)	0.389
4 vs. 1	1.58 (1.156–2.159)	**0.004**	1.235 (0.891–1.713)	0.205
5 vs. 1	1.957 (1.438–2.662)	**<0.001**	1.717 (1.234–2.389)	**0.001**
Clinical T stage				
T2a vs. T1c	0.886 (0.651–1.204)	0.438	0.922 (0.667–1.273)	0.621
T2b vs. T1c	1.049 (0.812–1.353)	0.716	0.991 (0.761–1.291)	0.947
T2c vs. T1c	1.527 (1.195–1.953)	**<0.001**	1.361 (1.047–1.769)	**0.022**
T3a vs. T1c	1.191 (0.867–1.636)	0.281	1.163 (0.834–1.621)	0.373
T3b vs. T1c	1.431 (0.977–2.097)	0.066	1.446 (0.965–2.165)	0.074
T4 vs. T1c	2.165 (1.065–4.402)	**0.033**	1.157 (0.532–2.518)	0.712
ECOG (1-2)	1.542 (1.257–1.892)	**<0.001**	1.194 (0.961–1.483)	0.091
RT modality (EBRT)	1.701 (1.336–2.164)	**<0.001**	1.212 (0.925–1.589)	0.163
mPSA [ng/mL^2^]	1.003 (1.001–1.005)	**<0.001**	1.002 (1–1.004)	**0.021**
NLR	1.113 (1.044–1.186)	**0.001**		
PLR	1 (0.998–1.002)	0.819		
LMR	0.952 (0.898–1.01)	0.101		
WBC [10^3^/μL]	1.084 (1.037–1.134)	**<0.001**		
LYMPH [10^3^/μL]	1.024 (0.916–1.145)	0.680		
NEUT [10^3^/μL]	1.121 (1.057–1.189)	**<0.001**		
EO [10^3^/μL]	1.842 (1.128–3.006)	**0.014**		
BASO [10^3^/μL]	1.166 (0.052–25.997)	0.923		
RBC [10^6^/μL]	0.684 (0.552–0.849)	**<0.001**		
HCT	0.957 (0.93–0.984)	**0.002**		
RDW	1.144 (1.054–1.242)	**0.001**		
PLT [10^3^/μL]	0.999 (0.997–1.001)	0.323		
PDW [fL]	1.013 (0.965–1.063	0.613		

HGB—hemoglobin concentration, AMC—absolute monocyte count, ISUP—International Society of Urological Pathology, RT—radiotherapy, EBRT—external beam radiotherapy, mPSA—maximum prostate-specific antigen concentration, NLR—neutrophil-to-lymphocyte ratio, PLR—platelet-to-lymphocyte ratio, LMR—lymphocyte-to-monocyte ratio, WBC—absolute white blood cell count, LYMPH—absolute lymphocyte count, NEUT—absolute neutrophile count, EO—absolute eosinophile count, BASO—absolute basophile count, RBC—absolute red blood cell count, HCT— hematocrit, RDW—red blood cell distribution width, PLT—absolute platelet count, and PDW—platelet distribution width.

**Table 3:** Cox Proportional Hazards Regression Analysis for freedom from distant metastases in patients treated with radiation therapy for localized or locally advanced prostate cancer.

Variable	Univariate Analysis		Multivariate Analysis	
	Hazard Ratio (95% CI)	*p*-Value	Hazard Ratio (95% CI)	*p*-Value
HGB [g/dL]	0.852 (0.745–0.974)	**0.019**	0.897 (0.78–1.031)	0.125
AMC [10^3^/μL]	2.119 (1.073–4.187)	**0.031**	1.409 (0.643–3.091)	0.392
Age [years]	1.017 (0.995–1.04)	0.131	1.015 (0.992–1.039)	0.205
ISUP Grade Group				
2 vs. 1	1.590 (1.073–2.355)	**0.021**	1.417 (0.945–2.124)	0.092
3 vs. 1	1.746 (1.036–2.942)	**0.036**	1.45 (0.853–2.464)	0.169
4 vs. 1	2.426 (1.441–4.084)	**<0.001**	1.81 (1.043–3.139)	**0.035**
5 vs. 1	3.648 (2.324–5.726)	**<0.001**	2.643 (1.607–4.346)	**<0.001**
Clinical T stage				
T2a vs. T1c	0.577 (0.301–1.106)	0.098	0.523 (0.256–1.072)	0.077
T2b vs. T1c	1.252 (0.798–1.962)	0.328	1.02 (0.626–1.659)	0.938
T2c vs. T1c	2.112 (1.148–3.145)	**<0.001**	1.651 (1.07–2.549)	**0.024**
T3a vs. T1c	1.361 (0.804–2.306)	0.252	1.02 (0.579–1.797)	0.946
T3b vs. T1c	1.657 (0.918–2.99)	0.094	1.318 (0.708–2.453)	0.383
T4 vs. T1c	2.769 (1.106–6.936)	**0.029**	1.186 (0.432–3.255)	0.741
ECOG (1-2)	1.555 (1.084–2.232)	**0.017**	1.138 (0.762–1.701)	0.527
RT modality (EBRT)	2.381 (1.566–3.619)	**<0.001**	1.649 (1.016–2.675)	**0.043**
mPSA [ng/mL^2^]	1.006 (1.004–1.008)	**<0.001**	1.003 (1–1.006)	0.055
NLR	1.035 (0.914–1.172)	0.586		
PLR	0.999 (0.995–1.002)	0.387		
LMR	0.923 (0.834–1.021)	0.120		
WBC [10^3^/μL]	1.065 (0.984–1.154)	0.121		
LYMPH [10^3^/μL]	1.075 (0.886–1.305)	0.463		
NEUT [10^3^/μL]	1.091 (0.982–1.212)	0.105		
EO [10^3^/μL]	0.413 (0.116–1.475)	0.173		
BASO [10^3^/μL]	1.123 (0.005–255.1)	0.967		
RBC [10^6^/μL]	0.782 (0.541–1.129)	0.189		
HCT	0.962 (0.917–1.010)	0.116		
RDW	0.988 (0.838–1.164)	0.884		
PLT [10^3^/μL]	0.998 (0.995–1.001)	0.230		
PDW [fL]	1.031 (0.954–1.115)	0.437		

HGB—hemoglobin concentration, AMC—absolute monocyte count, ISUP—International Society of Urological Pathology, RT—radiotherapy, EBRT—external beam radiotherapy, mPSA—maximum prostate-specific antigen concentration, NLR—neutrophil-to-lymphocyte ratio, PLR—platelet-to-lymphocyte ratio, LMR—lymphocyte-to-monocyte ratio, WBC—absolute white blood cell count, LYMPH—absolute lymphocyte count, NEUT—absolute neutrophile count, EO—absolute eosinophile count, BASO—absolute basophile count, RBC—absolute red blood cell count, HCT—hematocrit, RDW—red blood cell distribution width, PLT—absolute platelet count, and PDW—platelet distribution width.

## Data Availability

Anonymized data available on request due to privacy and ethical restrictions.

## Supplementary Materials

The following supporting information can be downloaded at: https://www.mdpi.com/article/10.3390/biomedicines10102514/s1, Table S1: Missing data percentage and median values of blood test parameters before and after Nearest Neighbor Imputation (k-NN); Table S2: Detailed data on irradiation of patients treated for localized or locally advanced prostate cancer; Table S3: Spearman’s rank correlation for pre-treatment morphology parameters in prostate cancer patients undergoing radiotherapy. The Spearman correlation coefficient with P-value below is given in each cell. Pairs where is no correlation are presented in red font; Table S4: Abbreviations.
