# Supplementary material for: Pre-Treatment Hemoglobin Concentration and Absolute Monocyte Count as Independent Prognostic Factors for Survival in Localized or Locally Advanced Prostate Cancer Patients Undergoing Radiotherapy
Source: Biomedicines. 2022 Oct 8;10(10):2514. doi: 10.3390/biomedicines10102514 (PMC9599626; doi:10.3390/biomedicines10102514)
Supplement: Supplementary file 1 [file biomedicines-10-02514-s001.zip › biomedicines-1944771-supplementary.pdf]

Supplementary Table S1. Missing data percentage and median values of blood test parameters before and after Nearest Neighbor Imputation (k-NN).

Parameter	Missing data (n)	Median (IQR) before k-NN	Median (IQR) after k-NN
NLR	31 (3.05%)	1.90 (IQR 1.41-2.64)	1.92 (IQR 1.42-2.62)
PLR	31 (3.05%)	114.6 (IQR 88.9-146.2)	114.8 (IQR 90.1-145)
LMR [$10^3/\mu L$]	31 (3.05%)	3.32 (IQR 2.57-4.31)	3.32 (IQR 2.57-4.28)
WBC [$10^3/\mu L$]	2 (0.20%)	6.43 (IQR 5.3-7.7)	6.43 (IQR 5.3-7.7)
LYMPH [$10^3/\mu L$]	31 (3.05%)	1.85 (IQR 1.48-2.36)	1.86 (IQR 1.5-2.35)
NEUT [$10^3/\mu L$]	14 (1.38%)	3.61 (IQR 2.87-4.56)	3.61 (IQR 2.87-4.56)
AMC [$10^3/\mu L$]	13 (1.28%)	0.56 (IQR 0.44-0.71)	0.56 (IQR 0.45-0.71)
EO [$10^3/\mu L$]	30 (2.95%)	0.14 (IQR 0.09-0.22)	0.15 (IQR 0.09-0.22)
BASO [$10^3/\mu L$]	15 (1.48%)	0.03 (IQR 0.02-0.04)	0.03 (IQR 0.02-0.04)
RBC [$10^6/\mu L$]	3 (0.30%)	4.48 (IQR 4.2-4.77)	4.48 (IQR 4.2-4.77)
HGB	0	N/A	N/A
HCT	16 (1.57%)	40.6% (IQR 38.6-42.9)	40.6% (IQR 38.7-42.9)
RDW	49 (4.82%)	13.3% (IQR 12.8-14)	13.4% (IQR 12.8-14)
PLT	0	N/A	N/A
PDW [fL]	243 (23.92%)	12 (11-13.4)	12.3 (IQR 11.2-13.6)

NLR—neutrophil-to-lymphocyte ratio, PLR—platelet-to-lymphocyte ratio, LMR—lymphocyte-to-monocyte ratio, WBC—absolute white blood cell count, LYMPH—absolute lymphocyte count, NEUT—absolute neutrophile count, AMC—absolute monocyte count, EO—absolute eosinophile count, BASO—absolute basophile count, RBC—absolute red blood cell count, HGB—hemoglobin concentration, HCT—hematocrit, RDW—red blood cell distribution width, PLT—absolute platelet count, PDW—platelet distribution width

Supplementary Table S2. Detailed data on irradiation of patients treated for localized or locally advanced prostate cancer.

Parameter	EBRT (n = 824) n (%)	EBRT + single BT-boost (n = 125) n (%)	EBRT + double BT-boost (n = 67) n (%)
Total dose [Gy]			
42 / 2	1 (0.12%)		
44 / 2	1 (0.12%)	1 (0.8%)	1 (1.5%)
46 / 2		1 (0.8%)	48 (71.6%)
50 / 2			17 (25.4%)
54 / 2		117 (93.6%)	1 (1.5%)
56 / 2		1 (0.8%)	
58 / 2	1 (0.12%)		
62.5 / 2.5	6 (0.72%)		
64 / 2		5 (4%)	
68 / 2	1 (0.12%)		
70 / 2	5 (0.61%)		
72 / 2	14 (1.7%)		
73.8 / 1.8	2 (0.24%)		
74 / 2	38 (4.61%)		
75.6 / 1.8	12 (1.46%)		
76 / 2	719 (87.3%)		
45 / 1.8 + 32 / 2	1 (0.12%)		
78 / 2	23 (2.8%)		
BT dose [Gy]			
10 / 10		108 (86.4%)	
10.5 / 10.5		17 (13.6%)	
14 / 7			1 (1.5%)
20 / 10			28 (41.8%)
21 / 10.5			37 (55.2%)
22 / 11			22 (32.8%)
Lymph nodes dose [Gy]			
0	199 (24.2%)	20 (16%)	25 (37.3%)
37.5 / 2.5	1 (0.12%)		
42 / 2	1 (0.12%)		
43.2 / 1.8	1 (0.12%)		
44 / 2	571 (69.3%)	103 (82.4%)	14 (20.9%)
45 / 1.8	20 (2.43%)		
46 / 2	9 (1.01%)	2 (1.6%)	14 (20.9%)
50 / 2	22 (2.67%)		14 (20.9%)
N_1 boost dose [Gy]			
60 / 2	4 (0.49%)		
64 / 2	1 (0.12%)		
66 / 2	6 (0.73%)		
70 / 2	1 (0.12%)		
72 / 2	1 (0.12%)		
75.6 / 1.8	1 (0.12%)		

EBRT—external beam radiotherapy; BT—brachytherapy

Supplementary Table S3. Spearman's rank correlation for pre-treatment morphology parameters in prostate cancer patients undergoing radiotherapy. The Spearman correlation coefficient with P-value below is given in each cell. Pairs where is no correlation are presented in red font.

	NLR	PLR	LMR	WBC	NEUT	LYMPH	AMC	EO	BASO	RBC	HGB	HCT	RDW	PLT	PDW
**NLR**		0.54<br><0.001	-0.65<br><0.001	0.24<br><0.001	0.62<br><0.001	-0.57<br><0.001	0.13<br><0.001	-0.21<br><0.001	-0.09<br>0.004	-0.02<br>0.427	-0.04<br>0.222	0.00<br>0.877	0.07<br>0.037	-0.01<br>0.811	0.03<br>0.329
**PLR**	0.54<br><0.001		-0.49<br><0.001	-0.35<br><0.001	-0.05<br>0.104	-0.76<br><0.001	-0.21<br><0.001	-0.24<br><0.001	-0.10<br>0.002	-0.19<br><0.001	-0.22<br><0.001	-0.20<br><0.001	0.00<br>0.9305	0.37<br><0.001	-0.21<br><0.001
**LMR**	-0.65<br><0.001	-0.49<br><0.001		0.00<br>0.878	-0.24<br><0.001	0.57<br><0.001	-0.51<br><0.001	0.09<br>0.005	0.07<br>0.038	0.05<br>0.100	0.04<br>0.157	0.00<br>0.875	-0.04<br>0.265	0.07<br>0.024	0.01<br>0.747
**WBC**	0.24<br><0.001	-0.35<br><0.001	0.00<br>0.878		0.87<br><0.001	0.58<br><0.001	0.60<br><0.001	0.23<br><0.001	0.25<br><0.001	0.24<br><0.001	0.24<br><0.001	0.27<br><0.001	0.12<br><0.001	0.30<br><0.001	0.04<br>0.208
**NEUT**	0.62<br><0.001	-0.05<br>0.104	-0.24<br><0.001	0.87<br><0.001		0.21<br><0.001	0.47<br><0.001	0.04<br>0.248	0.13<br><0.001	0.18<br><0.001	0.17<br><0.001	0.21<br><0.001	0.13<br><0.001	0.24<br><0.001	0.04<br>0.212
**LYMPH**	-0.57<br><0.001	-0.76<br><0.001	0.57<br><0.001	0.58<br><0.001	0.21<br><0.001		0.33<br><0.001	0.31<br><0.001	0.24<br><0.001	0.21<br><0.001	0.22<br><0.001	0.22<br><0.001	0.06<br>0.060	0.24<br><0.001	0.02<br>0.615
**AMC**	0.13<br><0.001	-0.21<br><0.001	-0.51<br><0.001	0.60<br><0.001	0.47<br><0.001	0.33<br><0.001		0.23<br><0.001	0.16<br><0.001	0.16<br><0.001	0.17<br><0.001	0.22<br><0.001	0.09<br>0.004	0.15<br><0.001	0.03<br>0.400
**EO**	-0.21<br><0.001	-0.24<br><0.001	0.09<br>0.005	0.23<br><0.001	0.04<br>0.248	0.31<br><0.001	0.23<br><0.001		0.37<br><0.001	0.07<br>0.036	0.05<br>0.113	0.04<br>0.205	0.05<br>0.100	0.09<br>0.004	0.12<br><0.001
**BASO**	-0.09<br>0.004	-0.10<br>0.002	0.07<br>0.038	0.25<br><0.001	0.13<br><0.001	0.24<br><0.001	0.16<br><0.001	0.37<br><0.001		0.12<br><0.001	0.12<br><0.001	0.09<br>0.003	0.21<br><0.001	0.22<br><0.001	0.18<br><0.001
**RBC**	-0.02<br>0.427	-0.19<br><0.001	0.05<br>0.100	0.24<br><0.001	0.18<br><0.001	0.21<br><0.001	0.16<br><0.001	0.07<br>0.036	0.12<br><0.001		0.83<br><0.001	0.85<br><0.001	0.05<br>0.097	0.03<br>0.269	0.07<br>0.027
**HGB**	-0.04<br>0.222	-0.22<br><0.001	0.04<br>0.157	0.24<br><0.001	0.17<br><0.001	0.22<br><0.001	0.17<br><0.001	0.05<br>0.113	0.12<br><0.001	0.83<br><0.001		0.92<br><0.001	-0.09<br>0.003	0.00<br>0.882	0.06<br>0.058
**HCT**	0.00<br>0.877	-0.20<br><0.001	0.00<br>0.875	0.27<br><0.001	0.21<br><0.001	0.22<br><0.001	0.22<br><0.001	0.04<br>0.205	0.09<br>0.003	0.85<br><0.001	0.92<br><0.001		-0.02<br>0.504	0.03<br>0.353	0.06<br>0.044
**RDW**	0.07<br>0.037	0.00<br>0.931	-0.04<br>0.265	0.12<br><0.001	0.13<br><0.001	0.06<br>0.060	0.09<br>0.004	0.05<br>0.100	0.21<br><0.001	0.05<br>0.097	-0.09<br>0.003	-0.02<br>0.504		0.08<br>0.010	0.13<br><0.001
**PLT**	-0.01<br>0.811	0.37<br><0.001	0.07<br>0.024	0.30<br><0.001	0.24<br><0.001	0.24<br><0.001	0.15<br><0.001	0.09<br>0.004	0.22<br><0.001	0.03<br>0.269	0.00<br>0.882	0.03<br>0.353	0.08<br>0.010		-0.29<br><0.001
**PDW**	0.03<br>0.329	-0.21<br><0.001	0.01<br>0.747	0.04<br>0.208	0.04<br>0.212	0.02<br>0.615	0.03<br>0.400	0.12<br><0.001	0.18<br><0.001	0.07<br>0.027	0.06<br>0.058	0.06<br>0.044	0.13<br><0.001	-0.29<br><0.001	

NLR—neutrophil-to-lymphocyte ratio, PLR—platelet-to-lymphocyte ratio, LMR—lymphocyte-to-monocyte ratio, WBC—absolute white blood cell count, LYMPH—absolute lymphocyte count, NEUT—absolute neutrophile count, AMC—absolute monocyte count, EO—absolute eosinophile count, BASO—absolute basophile count, RBC—absolute red blood cell count, HGB—hemoglobin concentration, HCT—hematocrit, RDW—red blood cell distribution width, PLT—absolute platelet count, PDW—platelet distribution width

Supplementary Table S4. Abbreviations.

AMC	absolute monocyte count
PCa	prostate cancer
HGB	hemoglobin concentration
IQR	Interquartile Range
WHO	World Health Organization
TAM	tumor-associated macrophages
OS	overall survival
FFDM	freedom from distant metastases
CT	computed tomography
MRI	magnetic resonance imaging
PET	positron emission tomography
PET-PSMA	positron emission tomography prostate-specific membrane antigen
ISUP	International Society of Urological Pathology
EBRT	external beam radiotherapy
BT-boost	brachytherapy boost
PSA	prostate-specific antigen
HR	hazard ratio
CI	confidence interval
AIC	Akaike Information Criterion
ADT	Androgen deprivation therapy
Neo-ADT	neoadjuvant androgen deprivation therapy
GnRH	gonadotropin-releasing hormone agonist
NSAA	nonsteroidal anti-androgen drug
ECOG	Eastern cooperative oncology group
NCCN	National Comprehensive Cancer Network
TURP	transurethral resection of the prostate
NLR	neutrophil-to-lymphocyte ratio
PLR	platelet-to-lymphocyte ratio
LMR	lymphocyte-to-monocyte ratio
WBC	absolute white blood cell count
LYMPH	absolute lymphocyte count
EO	absolute eosinophile count
BASO	absolute basophile count
RBC	absolute red blood cell count
HCT	hematocrit
RDW	red blood cell distribution width
PLT	absolute platelet count
PDW	platelet distribution width
mPSA	maximum prostate-specific antigen concentration
RT	radiotherapy

UVA	univariate analysis
MVA	multivariate analysis
